# A theory of the skill-performance relationship

**DOI:** 10.3389/fpsyg.2024.1296014

**Published:** 2024-02-09

**Authors:** Seppo E. Iso-Ahola

**Affiliations:** Department of Kinesiology, School of Public Health, University of Maryland, College Park, MD, United States

**Keywords:** skill, ability, talent, effort, practice, momentum, theory

## Abstract

The skill-performance relationship is a cornerstone of a meritocratic society. People are selected for schools, colleges and jobs based on the premise that more skillful individuals perform better. Scientific understanding of the skill-performance relationship demands that the effect of skill on performance is objectively assessed without subjective, social, and political considerations. One of the best areas for this analysis is sports. In many sports settings, the skill-performance relationship can objectively be examined at the technical, behavioral, psychological, and neurological levels. This examination reveals that skill and performance are inextricably intertwined. While skill affects performance, performance in turn defines and affects skill. To disentangle the previously confusing and interchangeable use of these key constructs, the paper presents a theoretical model specifying that ability and effort have their own direct effects on performance, as well as indirect effects on performance through skill possession and skill execution in cognitive and physical domains of human performance. Thus, ability and skill are not the same. Although skill is a key determinant of performance, recent theory and research suggests that successful performers are successful not just because of their skills per se, but because they take advantage of their skills by creating more occurrences of momentum, making them last longer, and using them to bounce back faster from streaks of unsuccessful performance. Thus, momentum is an important mediator of the effects of skill on performance.

## Introduction

Is human performance simply a matter of skill? If it is, skillful individuals would then perform better than less skillful ones. Most people would probably argue that it cannot be so simple because there are many other factors that can potentially affect performance. But even if there are other factors, skill could still be the most influential of them all, especially because its subcomponents include technical, physical, and mental skills, as well as neural correlates. Furthermore, regardless of other factors, advanced skills are necessary for high-level performance in any areas of human performance. All of this suggests that skill explains a substantial amount of variance in performance and raises a question about the two key determinants of skill, namely, ability (“talent”) and effort/practice.

This paper reviews the relevant research literature to disentangle the skill-performance relationship and advance theoretical understanding of the effect of skill on performance, and vice versa. The roadmap of the review is three-fold. In the first part, it shows an entangled web of the key constructs due to a loose usage of them in previous analyses, for example, equating ability with skill. It then seeks to clarify the confusion through a new theoretical model that spells out the relationships between ability, skill, effort, and performance. Research suggests that skill must be understood and measured at the technical, behavioral, physical, mental (cognitive), and neurological levels. Finally, the reviewed research makes a strong case for the moderated and mediated effects of momentum on the skill-performance relationship.

The paper is not concerned with talent-development models and programs and how giftedness develops and is developed into achievement and success through educational and other programs, as these topics have extensively been addressed in the research literature (e.g., Simonton, 1999; Winner, 2000; Tannenbaum, 2003; Sternberg and Davidson, 2005; Subotnik et al., 2011; Preckel et al., 2020); nor is the review directed at the question how people develop into expert performers (e.g., Ericsson and Lehmann, 1996; Ackerman, 2014; Ruthsatz, 2014). Naturally, some research from these areas is relevant for the present analysis. The focus, however, is on an explanation of performance and its determinants in various skill-demanding contexts, from pro athletes competing to amateur golfers playing for a 20-dollar bet to musicians auditioning for orchestral positions and students taking exams. Most performance situations involve social evaluative threats that arise when skills are publicly displayed and evaluated, achievement of central goals potentially blocked, and performers’ social status undermined (Rohleder et al., 2007).

## Objective vs. subjective assessment of skill

A scientific understanding of the skill-performance relationship presupposes that skill is assessed objectively. In most areas of human performance, however, skill is determined subjectively. For example, in competitive tournaments, judges subjectively assess ice skaters’ and ballroom dancers’ skills and performance (e.g., Rohleder et al., 2007). Similarly, supervisors often evaluate their workers’ skills and performance based on their personal views, even if they have objective criteria and data available for the task (e.g., Ivancevich and Lyon, 1977). All of this means that subjectivity greatly muddies the water and makes it almost impossible to understand and explain the true relationship between skill and performance. A scientific analysis of this relationship, therefore, calls for objective ways of measuring skill and performance (Fleishman, 1975). The best arenas for this analysis are those where subjectivity is completely removed, and the level of performance is objectively determined. Pilots’ and air traffic controllers’ “vigilance performance” is one example of tasks conducive to such analyses. Some sports (e.g., golf and tennis) also provide good avenues for objective determinations of effects of skill on performance.

The present paper focuses on a theoretical examination of the skill-performance relationship in performance contexts where this fundamental relationship is not a product of subjective, political, and social considerations. Although not being the center of the analysis, arguably, golf is one of the best activities for such an objective examination because a person’s performance is a pure quantitative score unmitigated by subjective factors, such as referees’ and umpires’ judgments being influenced by the red color of competitors’ clothing (e.g., Hagemann et al., 2008). Therefore, this sport is used throughout to illustrate the proposed theoretical underpinnings.

### Importance of skill-performance relationship

Understanding the skill-performance relationship is important for two major reasons. First, the idea of a meritocratic society is built on the skill-performance premise. Accordingly, people are evaluated based on their performance, which is assumed to reflect their skillfulness; thus, they are selected for jobs, schools and colleges based on the exhibited skills. The underlying assumption is that if individuals become increasingly more skillful, they will perform better and become more successful. This, then, justifies greater investment of time, effort, and money in improving one’s skills through more education, more training, more practice etc.

Success, and even survival, in today’s competitive and technologically fast-changing society requires a “growth mindset,” according to which people believe that skills can be developed and improved rather being fixed to individually constrained levels (Dweck, 2017; Yeager et al., 2019). Work organizations are increasingly adopting and encouraging this mindset focus with emphasis on continuous learning and reskilling their employees. The half-life of skills (the time in which a skill flourishes but then becomes irrelevant) continues to decline, dropping from 30 years to the present 6 years. An obvious implication is that continuous skill development is essential in modern society. Another implication is that as workers’ skills grow, their financial status improves, and careers become more successful.

Second, if people aim to become high-level performers, they need to know which skills to develop and hone to achieve exceptional levels of performance. Olympic ice-skating aspirants have to know and acquire not only the technical and physical skills, but also mental skills needed for high performance in this sport. In a similar vein, students must know their weaknesses and strengths in various academic skills so that they can improve their performance.

Understanding the skill-performance relationship is also important from observers’ perspective. Observers are often called on to make assessments on why some performers are better than others. In sports, for example, skill-performance information is critical for certain professionals (e.g., scouts) whose job is to evaluate and recruit young talents. Although much less consequential, spectators and “Monday morning quarterbacks” also evaluate athletes in terms of their skills, typically attributing great performers’ success to their “talent.” Accordingly, many argue that Steph Curry is better than others because of his talent. However, scientifically, such explanations of the skill-performance relationship are superficial and shallow, but not lacking entertainment value.

The problem with superficial analyses is that they lead to oversights and simplifications of the skill-performance relationship. Surprisingly, no psychological research has raised or analyzed the most fundamental question, from where is which inferred? As a result, the picture of the skill-performance is muddied and entangled.

1. *Is skill inferred from performance? or*
2. *Is performance inferred from skill?*

Yet, a third possibility is that the causal arrow goes both ways, from skill to performance *and* vice versa, suggesting that the two are inextricably interwoven and correlated. That skill and performance are not independent of one another means that skill affects performance and performance defines and affects skill. Therefore, the two constructs can be used neither interchangeably nor independently. It is important to note that skill is always observed, assessed, and inferred from performance, typically at the conclusion of performance. Because individuals always perform on their skills, the two cannot completely be separated from one another. That is, people cannot perform without some amount of skill, but at the same time, skill is not the sole determinant of performance either. Thus, skill does not exist as its own entity, as an isolated object that can be measured without performance. One cannot point to a skill like he/she can point to a hand or leg as its own thing. Skill is always manifested in performance, and performance is an indicator of skill. The better one performs, the better is his/her skill deemed to be.

Taken together, while performance defines skill and affects skill, skill in turn influences performance. This is not to suggest that skill is the only determinant of performance or not to imply that performance is the only determinant of skill but instead, to show the theoretical and logical interdependence between the two. The interrelationship between skill and performance must be considered in a broader context of other contributing factors, such as practice/effort and confidence and anxiety, as explained later. Because skill and performance are not fixed entities but temporary and variable qualities or entities, they form a dynamic and changing reciprocal relationship. For example, the variability of skill’s effect on performance becomes clear when considering the difference between *skill possession* and *skill execution*. Possession of a certain level of skill does not automatically lead to invariant performance at that level, because skill execution fluctuates as a function of how well or poorly individuals are able to use their skills during performance, for example, by creating psychological momentum or avoiding anxiety, as explained later.

### Loose use of concepts

To better understand the skill-performance relationship, it is essential to not only examine the effects of skill on performance but also the main determinants of skill, namely, ability and effort (practice). A major problem is that ability, skill, and performance have invariably been used interchangeably in the reported studies and analyses. For example, Franks and Goodman (1986) suggested that the skill of players can be quantified using performance measures; Swaap et al. (2014) defined ability as “consistent performance at very high levels”; and Yarrow et al. (2009) argued that “skill is a level of performance in any given task.” In his much- heralded paper, Ferguson (1954) defined ability as a “skill learned to a crude level of stability,” whereas Fleishman (1958) assessed ability by initial performance on an experimental task, self-ratings, or tenure.

Such loose conceptual uses of these key constructs can lead to erroneous and unjustified conclusions. A good example of this is a recent meta-analysis by Gullich et al. (2022) in which the authors audaciously claimed that their main predictor (childhood/adolescent multisport practice vs. early specialization) explains “what makes a champion” even though the study did not measure individual performance; instead, performance was defined as the level at which athletes compete and was determined in some cases by coaches’ subjective ratings. Naturally, individual performance and competition level are entirely two different things, especially when the purpose is to argue and explain who becomes a champion. Inexplicably, the study also failed to consider how the main predictor (early multisport practice) affected athletes’ chances of reaching world-class and national levels of competition through increased skill. Multisport practice is irrelevant unless it is known if and how this variable changed athletes’ skills and thus their performance. It should also be noted that the study’s chief conclusion (i.e., multisport practice correlated with the highest “senior” but not with “junior” competition levels) is a truism: naturally, senior athletes have had more time to benefit from competitive and multisport experiences, as well as from practice and continuous improvement of domain-specific skills, than juniors and therefore should reach higher levels of competition, whereas early specialization would obviously have greater effects on junior athletes in the first part of their careers. Moreover, “performance milestones” are different for seniors than juniors.

Crucially, evidence has shown that (negative) exponential function is better than power function in predicting performance ratings over years of practice and skill development, indicating that a great number of performers benefit substantially from a delay of several years (Gaschler et al., 2014; Vaci et al., 2019). In short, these kinds of major conceptual and methodological problems occur when performance and skill are not clearly defined and measured but are instead used interchangeably in empirical studies.

**Figure 1:**
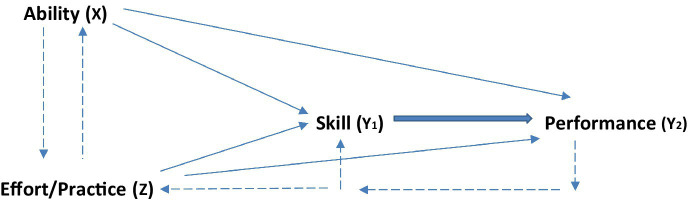
A theoretical model of determinants of skill and performance.

In the present analysis, ability, skill, effort, and performance are distinguished as their own constructs, and their interrelationships are delineated in a new theoretical model later (Figure 1). Ability refers to the innate capacity to understand and learn information for acquiring physical and cognitive skills. For example, acquisition of complex motor skills requires a capacity to coordinate body parts and implements used in performance. Thus, golfers must be able to coordinate their upper and lower body segments to swing a club smoothly. Similarly, the capacity to understand and learn math and linguistic concepts is the basis for cognitive skills (Hunter and Schmidt, 1996; VanLehn, 1996).

As for skills, they refer to learned or acquired physical and mental tools or qualities to execute certain actions required for successful motor and cognitive performance in specific domains. As such, skill can be thought of as a learned ability to perform at a specific level because a person has acquired the necessary mental and physical “know-how” for doing so. For example, a golfer must learn to hit iron clubs down into the ground to get the ball up in the air. For this to be a skill, however, h/she must be able to do it consistently (although not every time). If h/she gets the ball up in the air only occasionally, h/she does not yet possess this skill. A skillful golfer, on the other hand, not only gets the ball up in the air almost every time but has learned to regulate the ball’s flight trajectory on demand.

Regarding effort, performance in any physical and cognitive activity demands mental and physical resources. Thus, effort refers to employment of personal resources (e.g., “effort to time,” Duckworth et al., 2015) for practicing and improving one’s skills, typically manifested in frequency, duration, and intensity of the usage of these resources. Besides practicing one’s skills over long periods of time, effort can also denote expended effort *in* a specific performance situation. Usually, however, practice is thought of as long-term practice of skills, which of course includes effort, whereas effort is often considered in terms of the amount of strength and energy exerted in a present achievement situation. Ericsson et al. (1993) and Ericsson and Ward (2007) have shown that “deliberate” practice correlates with expert performance, even arguing that 10,000 h of this kind of practice leads to top-level performance in various domains. Relatedly, mental effort has been linked to the engagement of cognitive control and performance (Shenhav et al., 2017; Szekely and Michael, 2021). Finally, performance refers to objectively or subjectively measured performance in cognitive and physical tasks.

## Skill, effort/practice, and performance

Given that skill and performance are inextricably linked, albeit being distinct factors, how does one answer such a straightforward question as: Playing golf, one competitor shoots 76 and his/her opponent 84, is the former more skillful or is his/her better performance a direct result of his/her more frequent practices lately? The question is equally relevant for both pros and amateurs. In other words, how much of one’s motor performance (i.e., shown in a golf score in general or on a given day) is due to skill vs. effort (practice)? Furthermore, to what extent is one’s performance determined by effort during a day’s performance? Is the performer concentrating fully to the end without giving up? More generally, what determines high-skilled performance? Is it explained by “talent” or practice or even more generally, by nature or nurture? Based on a long line of research cumulated over the past 30 years, it has been argued that in most tasks, “deliberate practice” explains about 50% of the performance variance (Ericsson and Ward, 2007) while “talent” accounts for much less (e.g., 7% by working memory in the sight-reading performance in music; Meinz and Hambrick, 2010). An important point about practice is not just hours of it but meaningful (deliberate) practice with feedback provided by teachers and coaches (Ericsson et al., 1993).

These kinds of percentage comparisons, however, are misleading and inappropriate for several reasons. First, it has been shown that both deliberate practice and talent are necessary but insufficient alone for a high level of performance (e.g., Winner, 1998; Ackerman, 2014). Logically and mathematically, a performer’s present skill is a multiplicative function of ability and practice/effort, with their relative weights varying from one performance domain to another and from one performer to another (Simonton, 2001). This means that it is not possible to reach a high level of skill by means of ability alone or effort alone; conceptually and mathematically, neither can be zero as both are needed for the best performance. At the same time, the rise to the top is simply not possible without “profound” innate abilities (e.g., Ackerman, 1996, 2014; Lubinski and Benbow, 2000; Kell et al., 2013). Abilities matter and are “fundamental prerequisites for high achievement” (Subotnik et al., 2011).

Iddekinge et al. (2018) reported that ability, motivation, and the ability-motivation interaction explained 60.1, 30.5, and 9.4%, respectively, of the variance in job performance, and concluded that the interaction effect is “unimportant.” This, however, is fundamentally a wrong conclusion because both ability and effort are necessary for the best performance, and they interactively influence performance. Regardless of the additive or linear effects, the best performance does not occur without the joint effect of ability and effort. These researchers’ conclusion demonstrates how simple statistical (9.4%) results by means of percentage comparisons on performance can easily be misinterpreted. These researchers even argued that the results “change the conversation” about how ability and effort (motivation) should be considered in practical performance situations, a suggestion clearly unwarranted.

Second, skilled performance is in part an outgrowth of the interaction effect of genetics and environment (GxE) (e.g., Plomin, 1998; Rowe, 1998). Using a motor task, Fox et al.’s (1996) experiment demonstrated that the effect of heritability on early trials increased with practice (from about 55%), with its contribution trending higher throughout performance trials. At the same time, environment (practice) had a significant effect as the least gifted individuals acquired higher levels of skill and performed better by the end of the trials than the most gifted individuals in the early stages.

The mistake Ericsson and other environmentalists (e.g., Howe et al., 1998) make is to equate the environment’s (practice’s) “main” effect with the interaction effect of genetics and environment, or to ignore this interaction effect. That is, they argue that only meaningful practice matters, yet abundant empirical evidence has shown that performance is the result of both talent *and* practice/effort (e.g., Fox et al., 1996). The interaction effect is evident when children self-select themselves into different activities based on “innate action patterns” *and* early learning experiences. It is also seen when the process of “the successive hurdles” weeds out in each higher stage of performance those who are not sufficiently talented to move up to the next level of skill (Ackerman, 2014). While practice is needed, it alone is not sufficient for surpassing the next higher hurdle.

The interaction effect, however, does not mean that genetic influence is innate in the sense that abilities are hard-wired action patterns impervious to experience, and as Plomin (1998) has found, genetic factors affect people’s experiences. Ackerman (2014) and others have shown that those with “innate action patterns” in their early childhood gravitate toward certain types of skill-related activities and therefore spend more time practicing such activities and tasks and as a result, have more successful experiences than those who struggle with the same skill tasks. For the former, in motor activities, skill execution is relatively easy in terms of motor control and coordination, thereby affording rewarding and enjoyable experiences and further strengthening their initial interest in these kinds of skill-demanding activities (Preckel et al., 2020).

The bottom line is that because talent (ability) is manifested in stable interindividual differences in “developed and innate qualities” (Ackerman, 2014), it would be logically fallacious to argue that anyone can become a high-level performer in any activity if he/she only puts 10,000+ hours of deliberate practice into it, as Ericsson et al. (1993) and Ericsson and Ward (2007) have argued for decades. Talent matters greatly even if it only contributes 7% statistically to performance variance (Meinz and Hambrick, 2010). Thus, the percentage comparisons are mathematically and logically incorrect because talent cannot be zero. Individuals cannot achieve high-level performance in any area without a considerable amount of ability. If one multiplies 50% (practice effect) by zero, the total naturally is zero, that is, a person can work hard but if h/she has no talent, h/she will never reach high levels of performance. But this does not mean that both talent and effort cannot have their independent (“main”) effects in various performance domains; however, a key is how they interactively affect performance. The consequence of all of this is that stable individual differences exist and matter. It should also be noted that individual differences exist not only in the overall achievement but importantly, in the rate at which people learn skills (Ackerman, 2007; Stafford and Dewar, 2014).

Third, studies comparing these percentages are marred with methodological problems. A good example is Macnamara et al.’s (2014) meta-analysis in which deliberate practice explained only 1% of professionals’ performance variance. A closer inspection of the study’s methodology, however, shows that so-called professionals consisted of people like insurance agents, computer programmers, and soccer officials; and athletes included mediocre performers such as club-level and middle-aged runners, and novice bowlers. In short, percentage comparisons between different groups to show the relative importance of deliberate practice vs. talent become meaningless because of these kinds of conceptual and methodological problems and lapses. Thus, it is not useful to supposedly “make the novel prediction that achievement in the long run depends more on effort than talent” (Duckworth et al., 2015, p. 367). Not only has this been argued for decades but more importantly, such relative comparisons, whether in general or in percentages, are not helpful because both talent and effort are necessary for better performance.

Finally, it should be noted that 10,000 h of deliberate practice as the gate-opener for top-level performance is a flawed concept because 10,000 h is a sum of practice hours and skill (and talent). In most cases, the accumulation of practice hours improves skills. Thus, practiced hours and improved skill are interwoven, which then raises a question: Which is it that makes a 50% contribution to performance? Is it the mere hours or mere skills or mere ability or a combination of all? Since practice and skill are causally related in a reciprocal manner (practice improving skill and improved skill motivating more practice), it is not possible to separate the magnitude (percentage) of the contribution of skill vs. practice to performance variance, much less that of practice vs. ability. Obviously, ability is included in skill by way of its independent effect but also through practice as skill training over time enables innate abilities and genetic factors to contribute more to skill development via effort expenditure (e.g., Fox et al., 1996; Plomin, 1998; Zohar, 1998), leading to better performance. In short, there is nothing magical about 10,000 h of deliberate practice. A key is the level to which those hours take one’s skill. If the achieved skill is not sufficiently high, top-level performance is not possible no matter how many hours and years have been spent in practicing, a simple truth that has entirely been overlooked in research on expert performance during the last 30+ years.

Conceptually, the situation is further complicated by the fact that skill is embedded in both past and current performance. It is therefore not surprising that past performance at the elite level (“personal best”) is the best predictor of present performance and success, explaining over 80% of the total variance in performance (Marsh and Perry, 2005). In other words, those who have performed at a high level in the past are very likely to perform at that level in the future and are therefore very likely to succeed in competition. Conceptually, however, this is problematic because the same variable is employed to predict itself. That is, past performance includes past practice and skill, and present performance similarly includes past practice and skill.

It is, then, unclear how much of past or present performance is due to skill vs. practice. In golf, for example, a score for 18 holes is a performance score on a given day, but how much of it is attributable to effort (i.e., how often a person practices and plays per week) vs. skill? Further, how much of this skill is attributable to practice vs. ability? Besides their theoretical importance, answers to these questions are meaningful from a practical standpoint, because in golf, the United States Golf Association (USGA) has developed a system according to which a long-term average of the last eight single 18-hole scores (called “handicap”) is used as an indicator of one’s skill (ironically, called “playing ability”), and millions of players are assigned to different categories of skill for competition based on their handicap. But questions arise: Is handicap a valid indicator of skill or practice or a combination of both? What is the role of ability in it?

According to psychometric theory (Nunnally, 1978), the measurement of one variable (handicap) cannot prove validity for two different variables. As noted, the issue arises because the performance score is made up of two key components, skill and effort (past and present practice), not just one variable (skill). Moreover, one cannot become a skilled performer without effort or practice nor without innate ability (Fox et al., 1996; Gagne, 1998; Plomin, 1998; Rowe, 1998; Winner, 1998; Zohar, 1998; Simonton, 1999; Ackerman, 2014). In short, from both conceptual and measurement standpoints, given that skill and effort are inextricably interwoven, such metrics as “handicap” cannot be viewed as a pure indicator of skill or ability.

### “Playing ability,” skill, or consistency of performance?

Handicap is a good example of the intersection where skill, ability, effort, and performance meet. A golfer’s skill is not just a matter of motor ability because it is a multiplicative product of ability and effort in technical, physical, and mental areas of performance. This means that handicap is indicative of performance consistency at a certain level of skill over time and across situations. In short, skill is manifested in performance consistency or reduced variability (Ferguson, 1954). As skill increases so does performance consistency (e.g., Turvey et al., 1982). Compared to an individual with low or moderate skill, a person with high skill not only performs better but in a narrower range. Thus, skill sets upper and lower bounds for one’s performance, with a lower skill manifested in a wider range of performance.

To properly assess the determinants of performance, separate tests of ability, skill and effort should be administered. In the absence of such tests, skill is inferred from performance and the two constructs are then used interchangeably, rendering concepts like handicap nothing more than performance consistency or average performance over time. Furthermore, consistency of performance is also determined by technical, physical, and mental skills. Thus, a golfer must have a skill (and knowledge) not only to hit his/her clubs technically and physically correctly but also, a skill not to “choke” under pressure and in critical situations (Baumeister, 1984; Iso-Ahola et al., 2016). If handicap is defined as a pure “playing ability,” it would mean that all the internal (e.g., anxiety and concentration) and external (e.g., distractions) factors are constant. If so, “potential” or possible skill would seem a more appropriate term because it suggests that under the best of circumstances, a person is able to play at his/her ideal level of performance.

However, performance environments are seldom ideal and rarely free of internal and external impediments. For example, it is common for golfers to become quickly frustrated and thus give up (an internal factor) trying to do their best. After slicing their first tee ball to the woods, many recreational players exclaim: “It is going to be one of those days.” This means that the contribution of their current effort to performance declines considerably as they tend to give up when things do not go in the expected and hoped way. However, USGA’s handicap is based on the idea that players try their best every time they play and hit a shot. A question, then, is: When a player forfeits his effort on a given day, should s/he report the resultant inflated score and therefore artificially increase his/her handicap? It is well known that some golfers intentionally seek to inflate their handicap to increase their chances of winning in amateur competitions.

### Ability and skill are not the same thing

It is clear from the above conceptualization and considerations that ability and skill are not the same. Ability refers to a relatively stable “developed innate quality” (Ackerman, 2014) often called “talent,” whereas skill is a variable entity that is largely determined by the interactive effect of ability and practice/effort. It is important to stress that talent cannot merely be attributed to genetic effects because the innate action patterns *and* early successful experiences together begin separating young children by their differing interests in activities (e.g., sports vs. music). By school age, these differences become stable individual differences that persist later in life. To put it simply, 10,000+ hours of deliberate practice will not make everyone an expert baseball player.

Continued practice and successful performances provide skill-enhancing experiences, such as learning to perform better in critical situations. In contrast, domain-specific “innate” abilities are fixed in a sense that interindividual differences in them are stable and maintain the same rank order between individuals in childhood and adolescence, and they arise from a complex interaction of genetics and early social experiences (Ackerman, 2014). Although innate abilities are “developed qualities” and inherited “action patterns” that can, to a limited degree, be improved during narrow windows of opportunity in early years (Ericsson et al., 1993; Ericsson and Ward, 2007), they are stable traits that are observed in an individual’s standing relative to others in the short and long run. Thus, talent is a predominantly fixed and stable quality, whereas skill is a predominantly variable and temporary entity. Working memory is an example of ability whereas hitting a golf ball in the center of a club face is an example of a skill. Together with practice, ability determines one’s present level of skill (Figure 1), but skill cannot determine ability.

In short, skills can be improved but talent cannot be changed, at least not meaningfully, if at all. However, the rate of learning can be improved *within* individuals (Bryck and Fisher, 2012; Stafford and Dewar, 2014; Duckworth et al., 2015), but resultant changes will not alter a relative standing *between* individuals. It is also important to note that individual differences between individuals in *performance* can change because of the practice effects; as pointed out, ability is not the only determinant of performance. Someone with a high ability but little practice will lose in sport competition to another person who has a lower (yet sufficient) ability but practices hard.

Skills can also stagnate and thereby become stable when they are not continuously practiced and improved upon by deliberate practice. Yarrow et al. (2009) showed that in sports, automaticity in skill execution can be achieved at lower levels of skill, but if skills are not improved, automaticity becomes “more a false ceiling than a measure of excellence” (p. 588), and as a result, skills remain stagnant at lower levels.

In theory, then, skill is a multiplicative function of the degree of ability and the amount of practice or effort devoted to skill enhancement in various cognitive and physical domains of performance. Individual differences in the amount of time dedicated to deliberate practice influence achievement (Duckworth et al., 2015). Individual differences in the effort expended *in* a specific situation also influence achievement, as often seen in sport competitions; thus, effort is situationally determined.

In general, traits operate in a multiplicative rather than additive manner (Simonton, 2001). The multiplicative or interactive effect means that dedicated effort or practice over time will help one turn the underlying ability into an increased level of skill and competence (Iso-Ahola and Dotson, 2016), transforming potential talent in youth into outstanding performance in adulthood (Subotnik et al., 2011). It also means that high-ability individuals are more likely to benefit from personalized coaching and tutoring than those with lower abilities (e.g., Preckel et al., 2020). Experimental evidence further indicates that the best performers not only benefit from initially higher performance, but their rate of improvement is faster (e.g., Stafford and Dewar, 2014), a finding contrary to the idea that performance is simply a matter of the quantity of deliberate practice (Ericsson et al., 1993).

It then follows that ability and skill are not the same even though many researchers use these constructs loosely and interchangeably. For example, Gonzalez-Diaz et al. (2012) dubbed elite tennis players’ skill to perform well in important situations a “critical ability” even though this skill clearly is acquired through competitive experiences, thereby not being an ability. The confusion gets much worse when ability is referred to as both skill and performance in one and the same analysis, as Hambrick and Meinz (2011) did regarding sight-reading in piano playing.

### Importance of ability

Naturally, people cannot do much about their ability (or lack of it) because it is largely a stable entity, yet in theory, they can work hard to improve their skills and thus compensate to some extent for an initially low ability. However, research suggests that ability limits the effects of effort/practice on skills. For example, more practice in music does not correlate with better music skills and performance (Mosing et al., 2014), suggesting that a relative lack of musical ability restricts the beneficial effect of practice on skill development and performance advancement. Likewise, it is hard to imagine that anyone could become an outstanding singer without innate ability. In tasks requiring perceptual speed and psychomotor abilities (e.g., air traffic control), as well as in complex cognitive tasks, abilities are good predictors of performance after extensive practice has been considered (Ackerman, 2007). These findings suggest that it is more difficult to compensate for a relative lack of ability in certain activities, although they do not completely rule out some compensation with added hard work. However, theory of the path of least resistance predicts that people are less likely to engage in hard work when required effort increases (Iso-Ahola, 2022).

The importance of ability is further demonstrated when individuals are weeded out in the “successive hurdles” process, with the consequence of talent (ability) predicting increasingly better who will surpass the next higher hurdle and finally reach an exceptional level of performance (Meehl and Rosen, 1955; Ackerman, 2014). Logically, an inevitable consequence of the process of surviving successive hurdles is that performers become more similar in ability at increasingly higher levels of performance. In other words, since there are relatively minor differences in ability among elite performers, these performers can mainly distinguish themselves from others by skill-enhancing practice and effort, both physical and mental. Yet, it is the ability that made it possible for them to hurdle over to a next higher level in the process, and to ultimately reach the top-level, an important point overlooked by Ericsson and his associates (e.g., 2007).

In theory, ability can be separated from skill through performance on ability tests. For example, one’s cognitive ability is typically determined or assessed from performance on standardized cognitive tests (e.g., SAT). Similarly, one’s motor ability can be assessed from various perceptual speed-accuracy and cognitive psychomotor tests. This raises a question whether performance on such ability tests can be taken as an unequivocal indicator of ability. For example, it is well known that students spend large sums of money in tutoring services to advance their skill for taking the SAT test. Thus, their performance on this test is appreciably influenced by practice, which questions such tests’ validity to measure ability. It also raises a broader question whether a valid measurement of pure ability is possible in the first place.

In a similar vein, if we wish to determine the contribution of ability to physical performance, we may administer a basic perceptual-cognitive motor test (e.g., hand-eye coordination) and then determine how well it explains variance in motor performance on its own and relative to the contribution of the amount of practice dedicated to honing motor skills, though such research has not been conducted. It is also possible to determine one’s present skill level in sports like golf by administering a test for accuracy and distance of golf shots hit at a driving range. Such tests, however, would naturally include the effect of ability in it even if it mainly reflects a skill level achieved by an amount of practice. Typically, though, only skill tests are administered, which means that performance on such skill tests is comprised of both ability and effort (practice); however, the relative contribution of these two cannot be determined from a reported skill score. All the above theoretical considerations highlight the entangled web of the relationships between the key constructs. To disentangle the conceptual muddling, a theoretical model is proposed next to better elucidate determinants of human performance.

## A theoretical model

In all domains of human performance, technical, physical/cognitive, and mental skills on one hand and innate ability and effort/practice on the other, are required for the best performance. In cognitive tasks, such as math tasks, math thinking ability as well as technical math knowledge acquired through training critically determine performance. Similarly, in motor tasks, such as golf, abilities for motor coordination and the integration of multimodal information (proprioceptive, tactile, and visual), as well as acquired technical knowledge and physical skills, are required to hit the ball correctly and well. In addition to technical knowledge and physical skills in cognitive and motor areas of performance, mental skills are required for the best performance. “Mental” refers to cognitive skills that are acquired through training, such as concentration, anxiety control, relaxation, and visualization (Iso-Ahola and Hatfield, 1986; Iso-Ahola, 1992).^1^

While practice improves technical, physical, and mental skills (e.g., Ericsson et al., 1993; Abernethy et al., 1994; Ackerman, 2014), it also indirectly enhances ability’s effect on skill, for example, by reducing the negative impact of such factors as anxiety on one’s attempts to utilize his/her abilities maximally (e.g., Ramirez and Beilock, 2011). In this way, skill practice clears the way for abilities to have a greater effect on performance. Exerted effort also impacts one’s perceived ability to act in the environment, for example, when hikers grow tired after expending physical effort, targets start looking further away and perhaps beyond their abilities (Witt et al., 2012). Ability, in turn, affects practice and effort in that people like to practice skills and participate in activities in which they are good. The net result is that ability and practice together contribute to the development of skills in domains that require specialized training (Simonton, 1999). Based upon these considerations and the preceding conceptualization, the following theoretical model is proposed:

Accordingly, ability and effort have both direct and indirect effects on performance. As Figure 1 illustrates, both ability and effort have a direct or independent effect on performance without their effects on skill. Supporting ability’s effect, research has shown that intelligence predicts job performance (Schmidt and Hunter, 2004) and academic achievement well (Kuncel and Hezlett, 2007). The indirect effects occur when ability and effort exert their influence on skill, which in turn has a direct effect on performance. In accord with this model, ability and effort determine both skill possession and skill execution (i.e., manifested in performance). In other words, ability and effort enhance one’s level of skill (skill possession) and his/her execution of skill in general and in specific performance situations. The relationships are recursive in that performance affects skill and effort/practice, resulting in a feedback loop that either strengthens or weakens skill’s effect on performance, and as suggested, practice can indirectly affect ability by paving the way for ability’s effect, for example, through acquired experiences and removal of internal and external impediments (e.g., anxiety). The thickness of the arrow from skill to performance signals that this relationship is the strongest.

It is important to note that even though both ability and effort have their own direct effects on skill, they also affect skill interactively, because, as discussed above and shown by the arrows in Figure 1, the two influence one another. Similarly, regarding performance, in addition to their own independent effects, ability and effort also have some effect from one another, thereby adding to their interactive effect. Further, even though skill has a direct effect on performance, this effect naturally includes both independent and interactive effects of ability and practice/effort on skills.

The proposed model allows for the assessment of the contributions of ability and effort to the overall skill (and performance) in both absolute and relative terms. In this determination, however, performance (e.g., golf score) cannot be used as a proxy for skill because it would muddy the water; both must be measured separately. A valid determination of skill, in turn, calls for the measurement of cognitive and physical abilities on the one hand and mental (e.g., visualization) and physical effort (i.e., time spent practicing one’s physical/technical skills) on the other. One relevant example of ability is working memory (WM), which has been found to be an integral part of skill and performance in most cognitive (e.g., Bailey et al., 2018) and many physical tasks. In a similar vein, it has been shown that numerical (0.35), verbal (0.19), and visuospatial (0.13) abilities correlate significantly with chess skill (Burgoyne et al., 2016). Even in motor tasks and sports, cognitive abilities (e.g., WM) play an important role in building and strengthening one’s skill and performance (e.g., Vestberg et al., 2012). Relatedly, “embodied cognitions” confer cognitive benefits and lead to superior physical performance (e.g., Warburton et al., 2013), as they enhance action-perception skills (Raab and Araujo, 2019).

The positive effect of WM on performance is diminished if a performer has not acquired requisite knowledge and actions (i.e., skills) through practice to deal with such interfering factors as cognitive load (e.g., Eccles, 2006; Paas and Merrienboer, 2020). Another example of the interaction effect of ability and effort is provided by research suggesting that individuals’ attempt to control attention is driven by both task-specific effort demands and general cognitive ability, though more by the former (Irons and Leber, 2020).

### Linear or quadratic effects?

The model presented in Figure 1 is a path analysis of the direct and indirect effects that can be tested statistically by standard linear regression analyses. The model predicts the variables’ (X, Z) direct and indirect effects on skill, and these effects can be tested by regressing Y_1_ on X, Z and XZ. The quadratic effect would be tested by regressing Y_1_ on X, Z, XZ, Z^2^, and XZ^2^, with XZ^2^ giving the quadratic test or function. Similar statistical tests can be performed for determining the linear and quadratic effects on performance.

**Figure 2:**
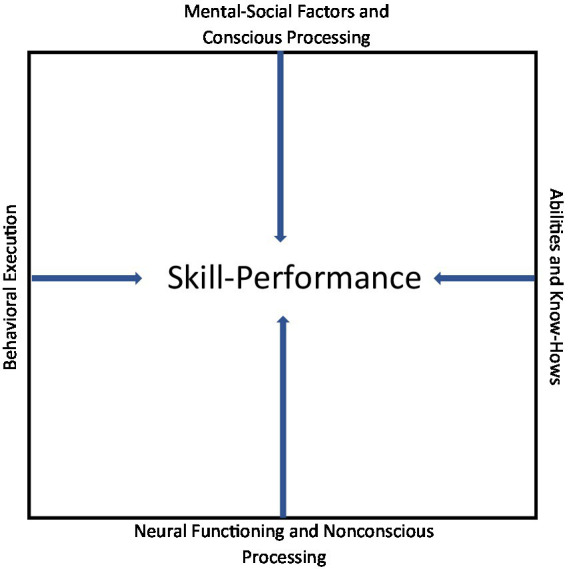
The four cornerstones of skill-performance.

Do ability and effort influence skill and performance in a linear or quadratic manner (skill’s effect on performance should only be linear)? A theoretical case can be made that ability’s effect would be linear, suggesting that the effect increases linearly even though it may reach an inflection point or a ceiling where the effect turns flat, and the positive effect becomes nonsignificant (for such an effect in team sports, see Swaap et al., 2014; Gula et al., 2021). What about effort? In this case, it may be theorized that the effect would be both linear and quadratic. That is, continuously increasing effort is good for skill and performance, but only up to a point after which it has no effect or even becomes negative (quadratic function). In music (Mosing et al., 2014), more effort does not translate into better performance. In sports, practicing technical or physical skills without working on mental skills is not likely to yield better results, or may even hurt performance. In golf, for example, one can hit balls at a driving range for 6 h every day, but if this practice does not include systematic honing of all aspects of skill (Figure 2), it is unlikely to improve the overall skill and performance. It remains to be determined not only how strong the effects of ability and practice/effort are, but also whether they are linear, quadratic, or both. It is also possible that these relationships can better be explained and predicted by exponential function when the time course of skill development and performance is considered (e.g., Gaschler et al., 2014; Vaci et al., 2019; Gula et al., 2021). This is a fruitful area for future research.

## The four cornerstones of skill-performance

As suggested, a skill is an acquired temporary and variable quality or tool (“know-how”) by means of which a person performs a task. Skill-performance rests on the four cornerstones (Figure 2): For example, a piano player must have acquired the necessary technical skills to perform correctly and well, but his/her successful behavioral execution of this skill in front of thousands of listeners or just a few friends is not guaranteed because it depends on conscious processing of relevant mental (e.g., concentration) and social (e.g., audience presence) factors. All of this is underpinned by neural functioning and nonconscious processing as neurons seek to optimize their performance and conserve energy because the activated circuits cannot work efficiently for long periods of time. Skilled performance can become automatic with enough repeats. In short, skill-performance is the result of the four elements or cornerstones coming synergistically together to produce the best outcome. The level of skill at which a person performs is the result of the combination of his/her talent, long-term practice, acquired know-hows, and effort put forth in behavioral execution of skills in a specific performance situation. Relevant research pertaining to these key elements is reviewed and discussed next.

### Abilities, know-hows, and behavioral execution

In cognitive and motor tasks, both technical possession of skills (e.g., the amount of relevant knowledge possessed) and behavioral execution of skills are needed for the best performance. In math tasks, for example, a person is more likely to be successful if he/she has a large repertoire of knowledge of relevant and basic math operations, as well as an ability to think mathematically using the acquired knowledge (Lubinski and Benbow, 2006). In a similar vein, a student writing a research paper not only needs to know the English grammar well, but he/she also must possess an ability to think cognitively and linguistically to be able to put sentences and paragraphs together into logically coherent wholes (VanLehn, 1996). In such motor tasks as golf, a player must have the knowledge of how to execute certain technical shots (e.g., chipping), as well as the behavioral skill of how to use this technical knowledge when playing an actual game. It is known that high-level performers have better technical, tactical, and procedural knowledge than less skilled performers (Elferink-Gemser et al., 2007, 2010).

Taken together, research suggests that performance variance is caused by a lack of proper technical knowledge, incorrect technical practice, and failed execution of skill. In golf, for example, hitting the ball requires a precise sequence of motor movements that must be learned to perform correctly and successfully. In the absence of the proper way of swinging, performance is highly variable and inconsistent, as seen with most recreational golfers. Unmastered skills lead to a more variable performance and worse outcomes. In contrast, skillful individuals perform with less variance (or in a narrower range of skill execution) and therefore achieve better results.

In most situations, performers must also have an ability to retrieve the necessary knowledge from its long-term and short-term memory storage (e.g., Norris, 2017; Artuso and Palladino, 2019). It has been demonstrated that the planning of a motor movement in one’s mind consists of retrieving a motor plan from the memory storage, and this retrieved plan has the neural signature similar to the actual execution of the plan (Mason and Just, 2020). If performance is not repeated often (e.g., golfers playing only once a month), knowledge is likely to decay, making the retrieval of the critical information difficult and deficient right when it would be needed for successful performance. If a particular technical skill is not often practiced, it becomes fragile and increasingly variable, and therefore hard to reproduce consistently. In contrast, research on “embodied cognition” has shown that memory of long and complex movement series in ballet can be enhanced by “marking” in practice, that is, by mentally rehearsing movement sequences rather than merely physically performing the entire program (Warburton et al., 2013). This movement reduction confers cognitive benefits (i.e., reduced cognitive load) and results in superior performance. The finding also demonstrates how closely cognitions and movements are related to one another (Raab and Araujo, 2019).

### Mental-social factors affecting behavioral execution

Behavioral execution of technical skills is associated with psychological factors that cause variation in performance. For example, in golf, recreational players often attempt to hit difficult shots that require considerably better skills than they presently have and as a result, typically can pull off such hard shots successfully only about 10% of the time. These attempts are often psychologically driven to make one look good and heroic in the rare case of being successful.

As this example illustrates, behavioral execution of a skill is significantly influenced by factors other than those dealing with the mere technical aspects of performance. One set of such factors is mental skills, which consists of, among other things, thinking clearly under pressure, not becoming anxious and nervous, concentrating well, and visualizing performance (Iso-Ahola, 1992, 1995). Deficiency in these skills leads to anxiety in critical situations, which reduces motor coordination and increases rigidity of movements, known as “freezing degrees of freedom” for movements, resulting in deteriorated performance (Bernstein, 1967; Marken, 1991; Berthouse and Lungarella, 2004). However, as a skill improves, additional degrees of freedom are unfrozen, making a young baseball batter’s swing increasingly more fluid and powerful (Turvey et al., 1982). In short, as performers become more skillful, they learn to regulate their degrees of freedom along with anxiety and other psychological disruptors (and enhancers).

In cognitive areas, many students experience anxiety when taking tests and therefore perform worse than their knowledge would predict. In a similar vein, due to stereotype threat effects, expert female chess players perform worse than male players on the same skill level (e.g., Smerdon et al., 2020). Thus, psychological factors often impair performance, yet they can also enhance performance when a person is well trained in mental skills and capitalizes on them for performance enhancement (e.g., Iso-Ahola, 1992; Schmid and Peper, 1998; Williams and Harris, 1998; Hatzigeorgiadis et al., 2011). Furthermore, competitors can improve their performance by accumulating experiences and utilizing them to enhance skills to perform better in critical situations. Evidence also indicates that psychological effects are stronger when competitors are equivalent in cognitive skills (Gonzales-Diaz and Palacios-Huerta, 2016).

One area of psychology that is particularly relevant for understanding the skill-performance relationship is conscious-nonconscious processing of mental operations. Evidence indicates that with expertise, skill execution becomes increasingly proceduralized and automatic because it is nonconsciously processed, with countless repeats resulting in improved performance (e.g., Yarrow et al., 2009; Moors, 2016; Bargh, 2017). On the other hand, if experts are made to consciously focus on skill execution, their performance worsens markedly, even dropping down to the level of novice performers (e.g., Gray, 2004). When performers’ conscious thoughts about performance are primed, these cognitions are likely to interfere with the well-rehearsed patterns of skilled movements, thereby turning smooth automatic actions into slower and more rigid movements with the net result of reduced confidence and ultimately worse performance. This is known as “choking,” and it is as common in cognitive as motor tasks (Baumeister, 1984; Lewis and Linder, 1997; Markman et al., 2006; DeCaro et al., 2011; Iso-Ahola et al., 2016).

Conscious interference with performance is also seen in the phenomenon known as “yips” (Smith et al., 2003; Papineau, 2015). Accordingly, performers with yips lose their skills, resulting in physical tremors and shaking of hands. The phenomenon has been known to seriously impede top performers’ motor actions in many sports from golfers to baseball pitchers and cricketeers. One major league pitcher recounted it in terror: “I do not know where the ball is going” (Naples, 2024). But the phenomenon is not limited to sports, as it has also been documented in young surgeons. Naples described how his “surgical yips” were caused by “a spiral of self-doubt in high-pressure situations demanding great precision.” However, he was able to overcome his yips and get his skills back, but only after a veteran surgeon admitted having made both technical and mental errors in his early years of performing surgeries. This veteran’s “authentic, warm, and open” advice was a critical social influence that helped Naples get rid of the yips and regain his skills to perform well.

In short, behavioral execution of skill does not occur in psychological or social vacuum but instead, is substantially influenced by internal and external factors. Confidence and anxiety are good examples of the former (e.g., Woodman and Hardy, 2003) and “social facilitation” (the effect of others’ presence on one’s performance) of the latter (e.g., Zajonc, 1965; Belletier et al., 2019). However, a more extensive analysis of the effects of such intrapersonal and interpersonal or contextual factors on skillful performance is beyond the scope of the present review, but can be found elsewhere (Iso-Ahola, 1995).

### Neural basis of skill-performance

Behavioral execution of skill is not only psychologically influenced but also neurologically based, as demonstrated by the experimental evidence showing that loss aversion correlates with performance declines (choking) and deactivation of ventral striatum at high levels of incentives in skilled tasks (Chib et al., 2012). Thus, the deactivation of ventral striatum appears to mediate the relationship between incentives and performance such that those with more stable neural activity, achieved by not focusing on possible failures, perform better in high-skilled tasks.

It is well established that neural preparation for action starts well before intention to act and the action itself (Libet et al., 1983a; Lau et al., 2007; Soon et al., 2008; Banks and Isham, 2009; Fried et al., 2011). Thus, unsurprisingly, skill execution is correlated with neuron-related factors, such as identification of neural representation of motor plans and encoding of movement sequences (e.g., Elbert et al., 1995; Penhune, 2013; Pilgramm et al., 2016). Both imagined and executed skill movements are not only neurally represented, but their neural signatures are similar (Mason and Just, 2020).

Evidence further suggests that the brain can activate various neural structures associated with action while blocking execution of that action (Hills, 2019). Besides such nonconscious cancelation of action, Libet et al. (1983b) argued that it is possible to consciously “veto” or stop internally generated actions, and Schultze-Kraft et al. (2016) showed that conscious cancelation is possible if it happens 200 ms before the movement onset. In other words, even though neural preparation for a certain action occurs before conscious intention, there is enough time to block that action altogether or select another motor plan for action execution. But it is unclear how the nonconscious brain processes and conscious thoughts interactively accomplish this vetoing task when there are only a few milliseconds to spare (Churchland et al, 2008; Haggard, 2011, 2019).

A skill does not exist in a specific neuron or a group of neurons, although performance variability of a group of neurons prior to the onset of a motor movement has empirically been linked to subsequent behavioral variability. Namely, Churchland et al. (2006) showed that over 50% of variance in behavioral performance was explained by neural performance prior to an initiation of the movement. This and other findings suggest that nonconscious operations start well before an actual execution of skill, and that the more a skill is practiced, the more it is relegated to nonconscious processing, resulting in greater efficiency in neural performance and fewer demands for cognitive control (Shiffrin and Schneider, 1977; Yarrow et al., 2009). In accord, evidence supports a relationship between performance and a neural marker (midline frontal theta, MFT) for the need for control such that better performance is associated with less MFT power (Pathania et al., 2019). This suggests that as performance improves, it becomes more automatic and therefore requires less cognitive control and corresponding neural engagement, with an adaptive performance environment being associated with reduced cortical activation (Miller et al., 2014).

In the process of skill acquisition and enhancement from the cognitive to associative to autonomous phase (Fitts and Posner, 1967), use of self-control is evident in early skill learning and leads to neuro-cognitive and task-relevant attentional engagement (Hunt et al., 2013; Jaquess et al., 2020). Behavioral training and practice facilitate a greater engagement of specific brain regions within the cognitive control network and increase the functional efficiency of the regions, thereby enhancing the capacity for cognitive control (Trommershauser et al., 2005; Chein and Schneider, 2012). However, the role of cognitive control generally declines with improved skill possession and skill execution.

Even if skill has neural substrates, it cannot be pinned down to a specific location in the brain, from which a linear causal chain of activity would commence. Rather, preparatory neural activity for voluntary action is believed to consist of interactive and iterative loops between several relevant areas (Pezzulo et al., 2014); in this process, the presupplementary-basal-ganglia-prefrontal cortex circuit converging on the primary motor cortex plays a critical role (Haggard, 2008). It is also known that certain brain areas and circuits are implicated in routine motor responses (e.g., a golf swing), and such responses quickly become automatic and “stubborn” (i.e., not easily changed) (Graybiel and Smith, 2014).

These findings suggest that skilled action is distributed across several regions and neural networks in the brain (Bilalic, 2018) and that automaticity can become a false ceiling for proficiency and skill (Yarrow et al., 2009). Following countless repeats, a person can reliably reproduce a skill at a certain level, but not necessarily at a high level. Repeating a bad golf swing can lead to automaticity as readily as repeating a correct swing. *But neurons do not know if the swing is good or bad.*

Skill has performance correlates in the brain such that continuous practice or application of a skill changes the brain structure and the neurocognitive basis of skill development, and thereby behavioral performance (e.g., Kelly and Garavan, 2005; Bezzola et al., 2011). Furthermore, extended practice promotes neural efficiency in that it takes less synaptic activity to produce internally generated sequences of movements (Picard et al., 2013). Improved perceptual motor skills and behavioral performance are associated with changes in the primary sensory cortex, among other things, in terms of neural tuning, spike synchrony and temporal response characteristics of neurons (e.g., Kilavik et al., 2009; Yarrow et al., 2009). Evidence also indicates that skill learning strengthens cortical representation of motor movements (e.g., Elbert et al., 1995; Baumeister et al., 2008; Wiestler and Diedrichsen, 2013; Yokoi et al., 2018). For example, it is known that practicing a golf swing increases the density of gray matter, which strengthens neural pathways relevant to this skill and attendant performance (Bezzola et al., 2011). Other factors being equal, the stronger the neural pathways have grown due to extended practice of a skill, the higher the skill possession and the better the skill execution.

Taken together, the reviewed evidence indicates that skills (and performance) have neural correlates in that neural performance correlates with behavioral skill execution. As neural performance improves and becomes more efficient, so do skill execution and behavioral performance. But as noted, this relationship is reciprocal because skills also influence performance, neural performance in this case. With continuous repeats, behavioral performance becomes more automatic, efficient, and less effortful, and as a result, neural functioning also becomes more efficient and less costly. In general, neural performance seeks to optimize and conserve its energy use as “a highly activated neural circuit cannot work efficiently for long periods of time due to a depletable pool of local resources” (Brzezicka et al., 2013). Similarly, synapses’ operation and functioning are based on the principle of resource optimization (Savtchenko et al., 2013) to manage or minimize neural fatigue in skill-requiring tasks, such as a chess marathon and 5-h rounds of competitive golf (Boksem and Tops, 2008). Continuous information processing has metabolic costs (Drugowitsch et al., 2012).

Although the present scientific understanding of the relationship between neural and behavioral performance is limited, this area promises to raise important questions for future research. One such question deals with the independent and interactive effects of neural (e.g., neural burden) and psychological (e.g., pressure) factors on human performance when controlling for the effects of skill. Neural burden is likely to rise when several motor programs in the brain compete for movement execution (Cisek and Kalaska, 2005; Churchland et al., 2008), with a possible consequence of impaired performance. In this regard, our research group is empirically addressing the following fundamental question: Do performers “choke” because of neural reasons (i.e., an old and discarded movement surfacing at a wrong time) or psychological reasons (e.g., pressure) or the interaction effect of the two?

## Momentum mediates skill’s effect on performance

What is it about increased skill that leads to better performance? Figure 2 suggests that those who have acquired a set of high technical, physical, behavioral, cognitive, and psychological skills underpinned by the efficiently functioning neural connections are better able to use these skills to their benefit. In this regard, it is important to note that the overall performance does not consist of a monolithic performance but instead, a series of separate performances. For example, in basketball and golf, total performance is comprised of many single runs of successful and unsuccessful performances (occurrences of momentum) *within* it.

Theory and evidence suggest that skillful performers make an initial success happen, which then forms the basis for psychological momentum in subsequent performance (Iso-Ahola and Dotson, 2014, 2016, 2017). Momentum is a psychological mechanism that has its roots in increased self-confidence, perceived competence and internal attributions brought about by an initial success, leading to a greater perceived likelihood of success (Iso-Ahola and Dotson, 2014, 2016). As momentum gains an upward spiral, its power to facilitate continued success increases, and becomes more nonconscious in the process, especially if performers’ skills have become proceduralized and automatic. It is well established that as sensorimotor skills improve, they become increasingly proceduralized and less conscious, thus not relying on explicit attentional control (e.g., Shiffrin and Schneider, 1977; Anderson, 1982; DeCaro et al., 2011).

**Figure 3:**
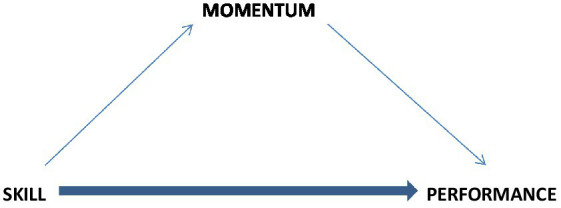
Momentum’s mediating effect.

A question, then, is about how the total performance is put together. Is it simply a sum of isolated performances here and there or patterned performances within? Evidence has shown that successful performers have a certain pattern to their performance, suggesting that momentum mediates the effects of skill on performance (Figure 3). This means that skillful performers have **more frequent** and **more lasting episodes of momentum** within their overall performance, and they **bounce back faster** from unsuccessful performance streaks (Iso-Ahola and Dotson, 2014, 2016, 2017). In other words, performers’ skills enable them to put together more runs of successful performances and ride them longer, as well as cut shorter the strings of unsuccessful performances. As a result of these momentum effects, skilled individuals are more likely to succeed when compared to less skilled performers, because, as noted above, skilled individuals are more able to create occurrences of momentum in the first place. When an initial success is of high intensity (e.g., a ferocious dunk), it gives rise to **high intensity momentum**, which is conducive to more frequent and more lasting episodes of momentum (Iso-Ahola and Dotson, 2014).

Momentum is psychological and behavioral in nature as it is subjectively (consciously or nonconsciously) experienced and behaviorally observed (e.g., Raab et al., 2012; Hubbard, 2015, 2016; Briki, 2017). These two facets of the phenomenon are intertwined in real-life situations such that initial success (behavioral outcome) is immediately followed by relevant perceptions, especially those related to competence, skillfulness, confidence, and momentum itself; these perceptions in turn form the basis for the perceived likelihood of further success and thus undergird momentum effects on performance and actual success, as evinced by a field study (Iso-Ahola and Blanchard, 1986).

In real life, as in fast-paced sports (e.g., basketball), performers’ perceptions go back and forth, fast and continuously, one feeding another so that a sense of momentum reinforces and strengthens perceptions of competence, confidence and likelihood of success, and vice versa, all of which leads to an upward spiral for more success. Because of the reciprocal relationships between these perceptions in fast-paced real-life situations, it is neither possible nor important to try to artificially create a frozen time in research labs to determine which comes first.

Perceptions, a sense of momentum, and actual performance operate in a continuous, iterative, and self-reinforcing loop until it is broken. Furthermore, in fast-paced situations, perceptions are largely, if not completely, nonconscious so that a sense of momentum is experienced without conscious awareness (Iso-Ahola and Dotson, 2016). In such performance contexts, confidence and skillfulness are experienced consciously only when momentum is lost and attention is turned to conscious efforts to get it back, often leading to a negative momentum and a spiral of deteriorating performance (e.g., Briki et al., 2013). When performance activity is not fast-paced (playing golf, vacuuming rooms etc.), conscious thoughts have more opportunities to interfere with the skill-momentum-performance loop and therefore to negatively affect performance. However, no studies have been undertaken to compare different performance situations in this regard, suggesting a fruitful area for future research.

It is axiomatic that good performance can be maintained only for limited time. In other words, momentum can sustain good performance for a relatively short time (e.g., Raab et al., 2012; Briki et al., 2013; Hubbard, 2015, 2016; Briki, 2017). Moesch et al. (2014) reported that short 5-min periods of momentum occurred in about 75% of all elite handball matches. Even the best basketball players make relatively few 3-point shots in a row. This is because competitive performances are not carried out in frozen situations and times where and when the effects of intervening variables are held constant or nullified. Furthermore, performers themselves get tired and anxious at various points in time throughout the overall performance, and opponents often block their skill-based performances. Thus, it is inevitable that good performance is composed of many short streaks of success within the overall performance.

In each competitive situation, performers’ skills are dynamic and variable due to internal and external factors affecting skill execution. Although this means that occasions of momentum-induced success are relatively short-lived, the number of them is critical for overall individual and team success. There is also evidence to suggest that if individuals or teams have a lasting streak of success going on *between* different performance days, such an enduring momentum may protect them from temporary losses and make negative momentums shorter *within* one performance day (Den Hartigh et al., 2016; Iso-Ahola and Dotson, 2017).

Even though skill is necessary for creating momentum, it does not automatically lead to momentum because (1) performers may not realize to use or be able to utilize their skills for building momentums and (2) because there are other non-skill related factors that can significantly contribute to the birth and loss of temporary momentums, such as competition between performers (Briki et al., 2013), time-outs called to stop the opponent’s momentum in basketball (Mace et al., 1992), and distractions in everyday tasks (e.g., making morning coffee, Botvinick and Bylsma, 2005; or driving in traffic, Iso-Ahola and Dotson, 2016). Nevertheless, in most performance situations, skill is essential for enabling performers to create momentum episodes and thus increase their chances of success. For example, an average golfer is not able to hit as many good 5-iron shots in a row as a skillful golfer, because he/she does not have sufficient skills for doing it. Amazingly, Steph Curry made 105 3-point shots consecutively in practice, no doubt thanks to his superior mental and physical skills.

Although skill and perceived momentum are positively related in theory, it is nevertheless possible to determine their independent and interactive effects on performance, as well as the possible moderating/mediating effect of momentum on the skill-performance relationship. Independent effects would indicate that skill and momentum have their own separate effects on performance, statistically indicated by significant “main effects” in an analysis of variance or by significant beta coefficients in regression analysis; a statistical comparison of beta coefficients pertaining to skill and momentum would show their relative effects in terms of the percentage of variance explained by each. An interactive effect, in turn, would suggest that skilled individuals are better able to use momentum to their advantage than unskilled individuals. Such an effect would signal a **moderating** effect of momentum.

In the case of a **mediating** effect of momentum on performance (Figure 3), momentum would significantly reduce the magnitude of skill’s effect on performance; in the strongest indication of mediation, the effect of skill on performance would diminish to zero when momentum is statistically considered (Baron and Kenny, 1986). A significant mediation effect would explain *why* skill affects performance, thereby supporting the proposed mechanism for the underlying effect (Iso-Ahola and Dotson, 2014, 2015, 2016). As no studies have directly tested the mediating effect of momentum on the skill-performance relationship, such research is suggested for future investigations.

According to the proposed model (Figure 3), skill is positively related to momentum, which in turn is positively related to performance. This last link was strongly supported by a large data set of over 11,000 observations derived from the PGA Tour pro golfers’ performance over four years (Iso-Ahola and Dotson, 2017). The data showed that the momentum indicators (frequency and duration of successful runs) explained between 85–92% of variance in performance outcomes of different intensity (i.e., top 10, 20, and 30 achievements). However, the link between skill and momentum was not directly addressed in the study but has indirectly been supported by other studies (Iso-Ahola and Mobily, 1980; Iso-Ahola and Blanchard, 1986). Taken together, the proposed model posits that the strength of the skill-performance relationship significantly depends on the moderating or mediating influence of momentum. It remains to be determined when and under what conditions, as well as in what areas of human performance, this mediating mechanism plays greater and smaller roles. Future research should also investigate whether momentum effects are linear or quadratic. Temporary effects of momentum suggest that the effects are both linear and quadratic, that is, positive momentum improves performance linearly, but at some point, the effect is likely to turn flat or downward into negative momentum (quadratic function).

In sum, skill, on its own, is an important determinant of performance, but this effect is further amplified when performers can put their skills to good use by creating momentum for performance. Improved skills enable and facilitate occurrences of momentum. In other words, skill has two effects on performance: a direct positive effect on its own and an indirect effect by creating and facilitating frequent and lasting occurrences of momentum, which in turn enhances performance. It then follows that successful performers are successful not just because of their skills *per se*, but because they are able to use their skills to bring about more instances of momentum, make them last longer and bounce back faster from streaks of unsuccessful performances.

## Summary and conclusion

Skill and performance are inextricably intertwined. Skill affects and is affected by performance, and performance defines skill and is determined by skill. Other things held constant, an individual or a team with greater skills will perform better, and successful and unsuccessful performances will impact the perceived level of skills and subsequent practice/ effort expended in improving skills. Because skill and performance influence one another, the causal relationship between the two is reciprocal and recursive. In general, human performance is a psychological process involving technical, physical, cognitive, behavioral, and mental skills undergirded by neural correlates in different domains of performance. Performance is critically shaped by skill (i.e., skill possession and skill execution), which in turn is determined by the independent and interactive effects of ability and practice (or effort). Thus, skill is a multiplicative product of ability and effort in cognitive and motor areas of performance because neither ability (talent) nor effort (practice) can be zero; both are necessary but not sufficient alone for the best performance. Comparison of the percentage contributions of each is therefore mathematically and logically misleading at best and incorrect at worst. It is theorized that ability affects skill and performance linearly up to an inflection point or a ceiling, whereas effort does so both linearly and quadratically. The empirical validity of this postulate remains to be determined.

A valid determination of skill’s effect on performance calls for objective measurements of mental and physical skills and abilities, as well as physical and mental effort and practice. It is important to note that ability and skill are not the same, even though they are often used interchangeably. Abilities are inherited action patterns that are manifested in stable individual differences in various activities by school age. In combination with effort/practice, abilities build skills, which are variable qualities subject to technical, physical, cognitive, and social influences. While prolonged practice improves skills, maximum effort in specific achievement situations is needed for the best performance. It is well established that less talented individuals (lower ability) are weeded out in the process of “successive hurdles” when they try to move up on the ladder to higher levels of performance, with the net result that performers become more similar in ability at higher levels of performance.

This conceptualization of skill and performance allows for the determination and assessment of the contribution of ability and effort in absolute and relative terms. As a direct determinant of performance, skill sets upper and lower bounds for performance, or a range of performance for a given level of skill. Skill is manifested in better performance and reduced performance variability. High-skill individuals not only perform better but in a narrower range. However, because skill execution can fluctuate as a function of such psychological factors as anxiety, one cannot always perform at his/her highest level of skills. Steph Curry arguably has the highest skill of making 3-point shots, yet his performance varies from one performance situation to another—either due to internal (e.g., concentration) or external (e.g., opponents) factors.

Studies have revealed that past performance predicts future performance exceedingly well. In other words, those who performed well in the past will perform well now and in the future. However, it is unclear from this general relationship what explains what, because both past and present performances include the contributions from skill, ability, and effort (practice or training). Thus, past skill is used to predict present skill or past effort is employed to predict present effort when past and present performances are compared. Such studies are not useful for understanding and explaining the effects of skill on performance.

Recent theory and research suggest that successful performers make better use of their skills in competitive events by creating opportunities for momentum. Thus, momentum becomes a psychological mechanism that further explains the underlying relationship between skill and performance, especially when considering that the overall performance consists of a series of single performances *within* it.

Is there a pattern to these single performances? Evidence has shown that skillful performers have more frequent and more lasting occurrences of momentum within the overall performance, and they bounce back faster from the streaks of unsuccessful performances. Furthermore, when an initial success is of high intensity, it is more likely to give rise to a sense of momentum, which then leads to more frequent and lasting episodes of momentum. All of this suggests a mediating effect of momentum on the skill-performance relationship such that skill increases momentum, which in turn positively affects performance. When the mediation effect occurs, the underlying skill-performance relationship is reduced after momentum is statistically considered. Thus, successful performers are successful not just because of their skills *per se*, but because they are able to use their skills to create more occurrences of momentum, make them last longer, and bounce back faster from streaks of unsuccessful performance.

## Author contributions

SI-A: Conceptualization, Writing – original draft, Writing – review & editing.

## Footnotes

^1^Although these are usually considered acquired mental skills, some of them can also be “natural” mental abilities, as in the case of an ability or an “innate action pattern” to thrive under pressure.
